# Cancer of the ampulla of Vater: analysis of the whole genome sequence exposes a potential therapeutic vulnerability

**DOI:** 10.1186/gm357

**Published:** 2012-07-04

**Authors:** Michael J Demeure, David W Craig, Shripad Sinari, Tracy M Moses, Alexis Christoforides, Jennifer Dinh, Tyler Izatt, Jessica Aldrich, Ardis Decker, Angela Baker, Irene Cherni, April Watanabe, Lawrence Koep, Douglas Lake, Galen Hostetter, Jeffrey M Trent, Daniel D Von Hoff, John D Carpten

**Affiliations:** 1 Integrated Cancer Genomics Division, Translational Genomics Research Institute, 445 N. Fifth Ave, Phoenix, AZ 85004, USA; 2 Virginia G Piper Cancer Center, 10290 N. 92nd St, Scottsdale, AZ 85258, USA; 3 Neurogenomics Division, Translational Genomics Research Institute, 445 N. Fifth Ave, Phoenix, AZ 85004, USA; 4 Clinical Translational Research Division, Translational Genomics Research Institute, 445 N. Fifth Ave, Phoenix, AZ 85004, USA; 5 Department of Surgery, Banner Good Samaritan Medical Center, 1111 E. McDowell Rd, Phoenix, AZ 85006, USA; 6 School of Life Sciences, Arizona State University, 427 E. Tyler Mall MC 5401 SOLS, Phoenix, AZ 85287, USA

## Abstract

**Background:**

Recent advances in the treatment of cancer have focused on targeting genomic aberrations with selective therapeutic agents. In rare tumors, where large-scale clinical trials are daunting, this targeted genomic approach offers a new perspective and hope for improved treatments. Cancers of the ampulla of Vater are rare tumors that comprise only about 0.2% of gastrointestinal cancers. Consequently, they are often treated as either distal common bile duct or pancreatic cancers.

**Methods:**

We analyzed DNA from a resected cancer of the ampulla of Vater and whole blood DNA from a 63 year-old man who underwent a pancreaticoduodenectomy by whole genome sequencing, achieving 37× and 40× coverage, respectively. We determined somatic mutations and structural alterations.

**Results:**

We identified relevant aberrations, including deleterious mutations of *KRAS *and *SMAD4 *as well as a homozygous focal deletion of the *PTEN *tumor suppressor gene. These findings suggest that these tumors have a distinct oncogenesis from either common bile duct cancer or pancreatic cancer. Furthermore, this combination of genomic aberrations suggests a therapeutic context for dual mTOR/PI3K inhibition.

**Conclusions:**

Whole genome sequencing can elucidate an oncogenic context and expose potential therapeutic vulnerabilities in rare cancers.

## Background

Advances in treatments for cancer have generally come incrementally because novel treatments are subjected to large prospective randomized clinical trials. In these studies, several hundred patients are randomized to one treatment arm or another and the treatment associated with the best outcome is advanced. This method has worked well for relatively common cancers, including breast and colon cancers. This approach, however, falls short when one is faced with rare cancers such that prospective trials involving large numbers of patients are difficult or impossible to conduct. In these cases, oncologists may choose chemotherapy regimens because the rare tumor is thought to be similar to a more common cancer for which an accepted standard treatment exists. Such is the case with cancers of the ampulla of Vater. These cancers account for only 0.2% of gastrointestinal cancers and approximately 7% of periampullary tumors. Periampullary tumors arise from either pancreatic ductal epithelium, the distal common bile duct, the duodenal mucosa, or the ampulla of Vater. When resectable, ampullary cancers are treated like pancreatic cancers with a pancreaticoduodenectomy. When they present at an advanced metastatic stage, there is little information guiding choices for chemotherapy regimens. Although they represent a minority in such trials, patients with ampullary cancers are often included in clinical trials of patients with biliary tract cancers, so these patients are often treated with gemcitabine and cisplatin [1].

Genomic technologies have resulted in some limited but remarkable advances in cancer treatment. Prior to the discovery of the Philadelphia chromosome and the identification of the BCR/ABL fusion protein leading to the development of imatinib, chronic myelogenous leukemia, a relatively rare form of the disease, was nearly uniformly fatal. Treatment was a bone marrow transplant with its attendant high risks of both morbidity and death. Treatment with imatinib, a tyrosine kinase inhibitor, can induce remission in approximately 87% of patients with greatly reduced risks of complications [2]. Imatinib was subsequently also found to be remarkably effective against gastrointestinal stromal tumors [3]. Other targeted drugs that have recently been shown to have efficacy in the setting of an indentified genomic aberration include vismodegib in advanced basal cell skin cancers harboring mutations in *PTCH1*, and vemurafenib in patients with advanced melanoma exhibiting a V600E mutation in the *BRAF *(v-raf murine sarcoma viral oncogene homolog B1) gene product [4,5].

The rapid advancement of genomic technologies offers the possibility to tailor chemotherapy based on an in-depth analysis of a limited number of tumor samples. The advent of next generation sequencing technologies has now paved the way for near complete interrogation of tumor genomes, providing the first opportunity for efficient global genomic tumor profiling at the point mutation, copy number, and breakpoint dimensions of the cancer genome. At a time in which there is an increasing array of chemotherapy drugs targeting aberrant molecular pathways, individualized genomic analysis to aid treatment decisions is quickly becoming feasible. Such an approach seems particularly well suited to the treatment of rare cancers for which there is a paucity of other clinical data to guide therapy. To demonstrate the potential clinical utility of individualized genomic analysis in patients with rare cancers, we applied whole genome sequencing to the tumor of a 63-year-old man with a resected cancer of the ampulla of Vater and identified therapeutic targets distinct from what would have been targeted based on existing literature.

## Materials and methods

### Samples

Written informed consent was obtained and the patient samples were collected for research purposes at Banner Good Samaritan Medical Center, Phoenix, Arizona. The study was approved by the Western Institutional Review Board (WIRB) and was conducted in accordance with the 1996 Declaration of Helsinki. This was a study entitled, 'Pancreas Cancer Biospecimens Repository' (WIRB® Protocol #20040832). Informed consent was obtained from the patient with cancer of the ampulla of Vater, including written consent for collection of the tissue and whole blood samples as well as clinical information and for genetic analysis of the specimens. The samples were then anonymized and assigned a unique identifier. Samples included fresh frozen tumor tissue collected within 20 minutes after surgical resection. Whole blood was obtained before the start of the operation at the time of induction of anesthesia. Histopathological analysis of the frozen specimen was quality assessed and determined to contain approximately 60% tumor cellularity. DNA and RNA were extracted from frozen tissue and whole blood using the Qiagen All Prep kit (Germantown, MD, USA) using the manufacturer's recommendations.

### Next generation sequencing

To facilitate whole genome next generation sequencing, we utilized the Life Technologies SOLiD™ (version 3) technology with mate-pair chemistry using the manufacturer's recommendations (Carlsbad, CA, USA). Briefly, 20 µg of genomic DNA is mechanically sheared to an average fragment size of 1.5 kb using the HydroShear. These size-selected fragments are then end repaired and circularized around a long mate-pair adaptor by nicked ligation. Nick translation is then used to displace the nick roughly 70 bp from either side of the internal adaptor. A nuclease reaction linearizes these fragments. SOLiD™ sequencing-specific sequencing adaptors are then ligated to the ends of these fragments. We prepared two independent 1.5 kb mate-pair libraries from the patient's constitutional (germline) DNA, and two independent mate-pair libraries from the patient's tumor DNA. Following PCR amplification, these mate-pair libraries are then used as templates in emulsion PCR reactions using SOLiD™ proprietary sequencing beads to generate clonal single molecule templated beads. Subsequently, an average of 500,000 templated beads are enriched and deposited onto SOLiD™ flowcells for massive ligation-based sequencing to generate 50 bp × 50 bp mate-pair sequences per bead. For this germline/tumor pair, we sequenced an average of one billion beads per library, thus generating two billion mate-pair reads for germline and two billion mate-pair reads for tumor.

### Next generation sequencing data processing

Raw next generation sequencing data in the form of csfasta and qual files are used to align 50 bp × 50 bp paired end reads from either the patient germline genome sequence or tumor genome sequence to the reference human genome (NCBI build 36, hg18). For alignment, we utilized the Life Technologies BioScope version 1.3 software suite, which is based upon a seed-and-extend algorithm [6]. Compressed binary sequence alignment/map (BAM) formatted output files for germline and tumor genome alignments are generated and PCR duplicates are subsequently removed using the Picard Tools.

### Next generation sequencing data analysis

#### Somatic single nucleotide variants

We employed two different algorithms. The first algorithm (SolSNP) [7] detects a SNP variant by comparing two discrete distributions. It compares the distance of the discrete sampled distribution of the base-pair pileup on each strand to the expected distributions (according to ploidy), and determines the genotype call. This is done using a Kolmogorov-Smirnov-like distance measure based on both the base (that is, reference or alternative base) as well as the confidence in the base called (that is, the quality score of each base in the pileup). If the genome is haploid, two expected pileups are created at each position: one consisting of only the reference base (a 'homozygous-reference' pileup) and another consisting of only the alternative base (a 'homozygous non-reference' pileup). The confidence of each pileup position is kept the same. The expected pileup that has the minimal Kolmogorov-Smirnov distance to the sampled pileup is considered to be the genotype of the locus on the strand. In diploid genomes, SolSNP also considers a pileup half of which is made up of the reference bases and the other half made of alternative bases (a heterozygous pileup). A locus on the chromosome is called a SNP if a variant genotype (either 'homozygous non-reference' or heterozygous) is detected on both strands. SolSNP can restrict its calls to loci where the genotype calls on both strands are identical. This is achieved by passing the 'Genotype Consensus' value to the parameter 'STRAND_MODE'. In this mode, the tool is able to produce genotype calls as well as variants. The second algorithm (Mutation Walker) calculates a test of proportions for the tumor/normal set to construct a test-statistic for reads in the forward direction and the reverse detection separately. The minimum of these two comparisons is used as the reported test-statistic, ensuring evidence is found in both the normal and reverse detection. Sites with evidence in the normal are filtered from the final report so as to reduce false positives arising from under-sampled polymorphic germline events. Calls common to both the algorithms were considered for further examination. To reduce the false negative rate, two sets of common calls were made. One was made with a strict and the other with a lenient set of parameters for both the algorithms. Both the sets were visually examined for false positives, which were then filtered to get a final list of true single nucleotide variants.

#### Indel detection

For detecting somatic indels we employed a two-step strategy. In the first step, we removed reads from the tumor sample BAM whose insert size lay outside the interval (500,5000) for SOLiD™. Genome Analysis Toolkit [8] was then used to generate a list of potential small indels from this BAM. A customized perl script, which used the Bio-SamTools library from BioPerl [9], then took these indel positions and for each of the indels looked at the region in the germline sample consisting of five bases upstream of the start and five bases downstream of the end of the indel. An indel was determined to be somatic only if there was no indel detected in the region under consideration.

#### Structural variants

Structural variants were analyzed by comparing two sources of information: relative normal/tumor read-level coverage and anomalously mapping read pairs. Assessing structural variants by read-level coverage is termed copy-number analysis since it is parallel in concept to microarrays. In copy number analysis, gains and losses were determined by calculating the log2 difference in normalized coverage between tumor and germline. Briefly, we investigated regions in 100 bp windows where the coverage in the germline was between 0.1 and 10 of the mode coverage in order to remove regions with high degrees of repeat sequence (for example, centromes or difficult to sequence regions. Normalized coverage was determined by the log2 coverage within a 100 bp bin over the overall modal coverage. We then reported the difference between the germline and tumor normalized coverage by a sliding window of size 2 kb. Deleted and amplified regions were flagged by a departure of greater than 0.75 from baseline. Moderate deletions were identified by a similar method utilizing sequence coverage rather than clonal coverage for consensus coding sequence exons only.

In anomalous read-pair analysis, we used perl scripts to detect enrichment of anomalously mapping read-pairs. These would be read-pairs that deviate from the expected mate-pair orientation of both reads occurring in the same direction or read-pairs that are outside the expected 1.5 kb insert size. A series of customized perl scripts were employed in the detection of translocation. These scripts used SAMtools [10] internally to access the BAM files. The analysis itself was made up of two steps. The first was the detection of a potential translocation in both tumor and germline samples. The second was comparison of a potential translocation in tumor to those detected in the germline sample to weed out potential false positives for statistical identification of outliers. The genome was analyzed by a walker with step size equivalent to the insert size where the number of anomalous reads was counted, that is, those reads whose mates align on a different chromosome. For each window we chose the highest hit to be the chromosome to which mates of most of the discordant reads mapped. We compared the ratios of discordant reads to the total aligned reads across all the windows to detect potential outliers. Outlier detection was done under the assumption that the normal distribution of the proportion of hit discordant reads in 2 kb windows aggregated across the chromosome will follow a normal distribution. We then computed the mean of the distributions and chose a cutoff of 3 standard deviations. The window with a proportion of hit discordant reads higher than this cutoff contained the region of potential translocation. The actual region of translocation is then determined by the span of the hit discordant reads in the window. For somatic translocations, the germline and the tumor sample are called separately and regions of overlap are eliminated. The output is a general feature format (gff) file of paired lines where the source tag indicates which two genomic regions show potential translocations. These regions were further inspected to reduce false positives and arrive at the more confident list. Additional details related to the methods for detection of somatic translocations and intrachromosomal rearrangements are included in Additional file 1.

### Validation of next generation sequencing findings

Briefly, ten single nucleotide variants and one local deletion were selected at random for chain termination sequencing (Sanger method). Validation was conducted using tumor DNA. Specific genomic primer pairs (Additional file 2) were designed to anneal in flanking single nucleotide variant regions and approximately 150 to 500 bp fragments to be amplified in 25-cycle PCR. Some primers carried M13 sequences on the 5' end as a back up for sequencing runs. Reaction products were column purified using a QIAquick PCR Purification kit (Qiagen) and submitted to the Arizona State University sequencing facility. Electropherograms were then manually examined for the presence of mutations/deletions in both orientations (Additional file 3).

Genomic quantitative PCR was performed to validate homozygous *PTEN *(phosphatase and tensin homolog) deletion (Additional file 4). In addition to the *PTEN *locus, genes located in adjacent regions of hemizygous deletion (*RGR *(retinal G protein coupled receptor) and *HHEX *(hematopoietically expressed homeobox)) were also measured. *BICC1 *(bicaudal C homolog 1 (Drosophila)) and *TRUB1 *(TruB pseudouridine (psi) synthase homolog 1), located in unaffected regions of chromosome 10, were used as internal controls. Quantitative PCR reactions were set up in a 384-well plate in triplicate with 3 ng of genomic DNA input per reaction. Amplifications were performed using a LightCycler480 instrument and SYBRGreen I Master Mix (Roche). Melting curves were examined for the presence of a single peak and Ct values were used in calculating fold-change according to the C_T _method [11]. All tumor and normal C_T _values were first normalized to glyceraldehyde 3-phosphate dehydrogenase (*GAPDH*). The quantity of genomic material present for each gene in the tumor sample was then normalized to its normal counterpart.

## Results

The patient is a 63-year-old Caucasian man diagnosed with adenocarcinoma of the ampulla of Vater. The patient had a Whipple procedure to resect the head of the pancreas, distal stomach duodenum, distal common bile duct, and gallbladder. The maximum dimension of the tumor, which was present at the junction of the ampullary and duodenal mucosa was 1.5 cm. The tumor invaded into the duodenal muscle wall but no lymphatic or vascular invasion was noted. There was no evidence of neoplasm of the lines of resection and there was no evidence of metastatic carcinoma to the 16 peripancreatic lymph nodes examined microscopically (pathologic TNM (Tumor, Node, Metastasis) stage T2, N0, M0). The patient's past history is significant of having smoked one to two packs per day for 15 years, stopping approximately 16 years before the diagnosis of his adenocarcinoma of the ampulla of Vater.

Massively parallel whole-genome sequencing was performed on genomic DNA from germline and tumor samples using the Life Technologies SOLiD™ version 4.0 mate-pair chemistry. Basic sequence run statistics based on our analysis pipeline are provided in Table 1. A total of 2.38 and 2.21 billion uniquely mappable reads were generated from germline and tumor DNA, which equates to 108 Gb and 100 Gb of uniquely mappable sequence for germline and tumor, respectively. Therefore, we achieved 37× and 40× genome coverage for tumor and germline, respectively. We detected a total of 2,771,201 SNPs from the germline genome, 91% of which are present in dbSNP (release 129). The transition to transversion ratio was 2.12, which is inline with what would be expected in a diploid human genome [12]. The full genome has been deposited in the database of Genotypes and Phenotypes (dbGaP) of the National Center for Biotechnology Information (submission ID SRA 053213).

**Table 1:** Basic sequencing statistics

Genome	Number of uniquely mapping 50 bp reads	Number of uniquely mapping bases	Genome coverage	Number of germline variants	Uniquely mapping tag pairs	Read-pair coverage
Germline	2,383,981,557	108,322,420,859	40	2,771,201	887,285,914	443
Tumor	2,215,368,333	100,400,536,852	37	-	863,886,211	432

Read pair coverage = (Number of uniquely mappable tag-pairs) × (Insert length)/Haploid genome size.

**Figure 1:**
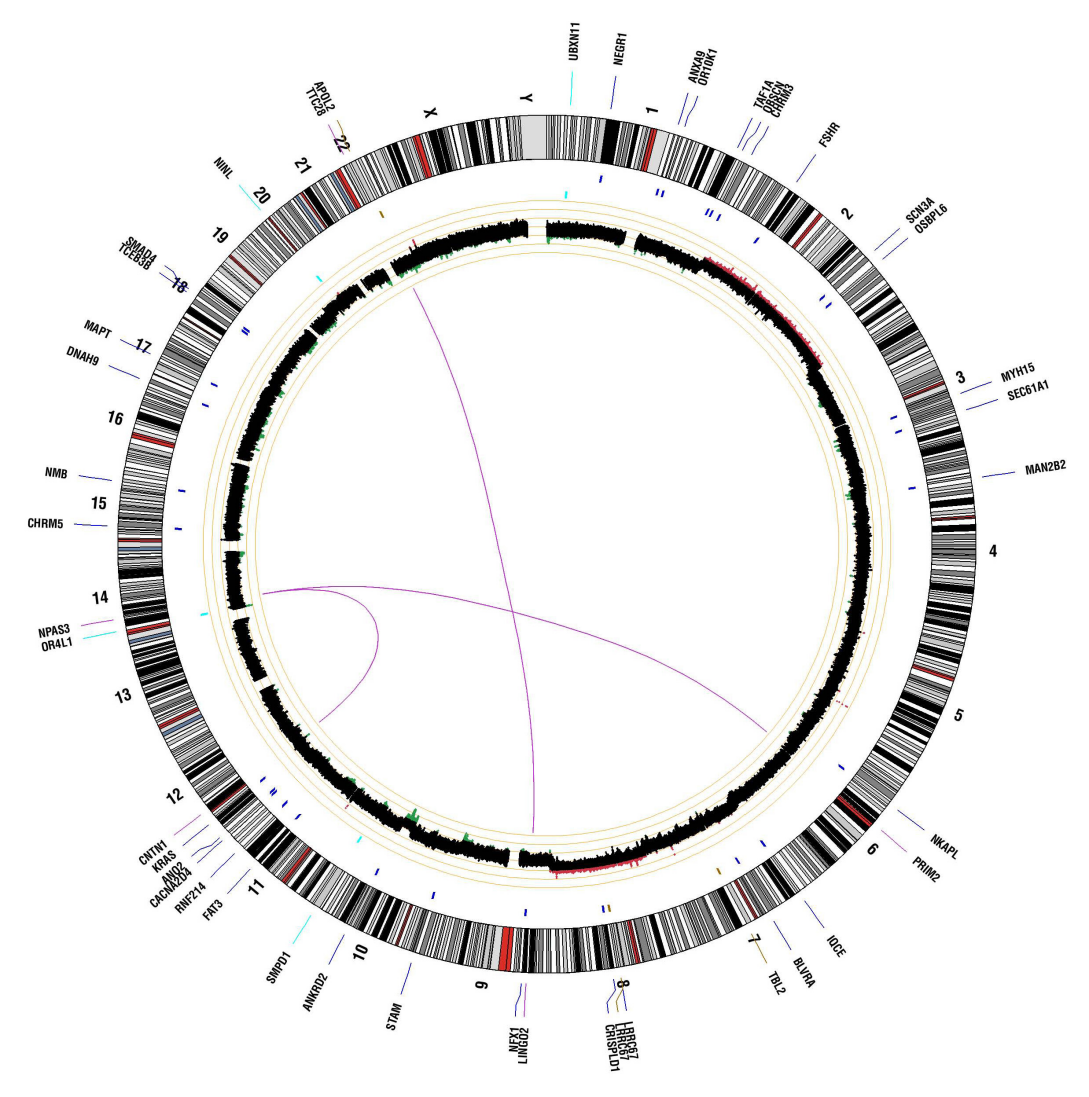
**Circos plot summarizing somatic events contained within pancreatic tumor of the ampulla of Vater**. The outer ring shows gene symbols for those genes somatically altered in the tumor relative to their map position against the human genome chromosome karyotype. Blue tick marks denote genes containing nonsynonymous point mutations. Cyan tick marks denote genes containing coding indels. Magenta tick marks represent discordant read pairs supporting putative translocation events and those genes involved in breakpoints. The inner ring represents somatic copy number events with regions of gain shown in red and regions of loss shown in green, with brighter colors denoting higher degrees of gain or loss. Magenta lines in the center represent breakpoint regions for translocation events.

To discover somatic mutations within ampullary cancer, we used a custom paired analysis pipeline. The overview of somatic alterations within this tumor is provided in the form of a Circos plot (Figure 1). Our paired analysis revealed 19,143 genome-wide somatic point mutations, of which 30 map within known annotated coding sequences. A list of all somatic missense (n = 28) and nonsense mutations (n = 2) is provided in Table 2. The most notable mutation is an activating *KRAS *(Kirsten rat sarcoma viral oncogene homolog) mutation at codon 12 (G12V), which is one of the most commonly reported mutations in ampullary carcinomas [13,14]. Furthermore, we discovered three somatic small insertions and deletions within coding regions, which result in frameshift mutations (Table 2). All missense mutations were assessed for likely functional consequences using the SIFT prediction algorithm [15,16], which characterized mutations as tolerated or damaging. Of the 28 missense mutations that were assessed, 19 (68%) were predicted to be damaging. Previously, we calculated the rate of SIFT damaging calls from a random set of approximately 10,000 missense variants from the 1000 Genomes data, which showed a rate of damaging mutations of 15%. Validation by Sanger sequence analysis is presented in Additional file 3.

**Table 2:** List of somatic coding point mutations and small indels

Genomic position and alleles^a^	Gene ID	Codon change	Amino acid consequence	SIFT prediction
Chr1:71831141G/A	*NEGR1*	503C>T	T168I	Damaging
Chr1:149227247C/T	*ANXA9*	760C>T	R245C	Damaging
Chr1:156702726C/A	*OR10K1*	751C>A	H251N	Damaging
Chr1:220801358G/T	*TAF1A*	1211C>A	A404D	Damaging
Chr1:226621463T/G	*OBSCN*	19592T>G	F6531C	Not scored
Chr1:238137913G/A	*CHRM3*	539G>A	R180Q	Damaging
Chr2:49235000G/A	*FSHR*	61C>T	R21W	Tolerated
Chr2:165735207G/C	*SCN3A*	362C>G	A121G	Damaging
Chr2:178905886C/T	*OSBPL6*	529C>T	R177X	-
Chr3:109658364C/T	*MYH15*	2137G>A	G713R	Damaging
Chr3:129258301T/G	*SEC61A1*	280T>G	L94V	Damaging
Chr4:6670038G/A	*MAN2B2*	2732G>A	R911H	Tolerated
Chr6:28336272A/G	*NKAPL*	1144A>G	S382G	Damaging
Chr7:2599270C/T	*IQCE*	1333C>T	R445X	-
Chr7:43813258G/A	*BLVRA*	790G>A	G264S	Tolerated
Chr8:76091857G/T	*CRISPLD1*	950G>T	C317F	Damaging
Chr9:33301117G/A	*NFX1*	1240G>A	G414R	Damaging
Chr10:17777173T/C	*STAM*	364T>C	Y122H	Damaging
Chr10:99328071C/T	*ANKRD2*	274C>T	R92W	Damaging
Chr11:6368506-6368518del	*SMPD1*	102-114del	L35WfsX72	-
Chr11:92170718A/T	*FAT3*	4894A>T	M1632L	Not scored
Chr11:116661033A/T	*RNF214*	2068A>T	T690S	Not scored
Chr12:1854754C/T	*CACNA2D4*	1730G>A	G577E	Damaging
Chr12:5902149C/A	*ANO2*	93G>T	Q31H	Damaging
Chr12:25289551C/A	*KRAS*	35G>T	G12V	Damaging
Chr14:19598288-19598307del	*OR4L1*	245-264del	I82TfsX104	-
Chr15:32142462G/A	*CHRM5*	252G>A	M84I	Damaging
Chr15:83002258G/C	*NMB*	130C>G	H44D	Tolerated
Chr17:11543819G/A	*DNAH9*	4919G>A	R1640Q	Tolerated
Chr17:41416912C/G	*MAPT*	905C>G	T302R	Damaging
Chr18:42815392C/T	*TCEB3B*	242G>A	R81Q	Tolerated
Chr18:46845916C/T	*SMAD4*	1081C>T	R361C	Damaging
Chr20:25405049-25405055del	*NINL*	2872-2878del	W958HfsX960	-

^a^Reference allele/mutated allele.

**Figure 2:**
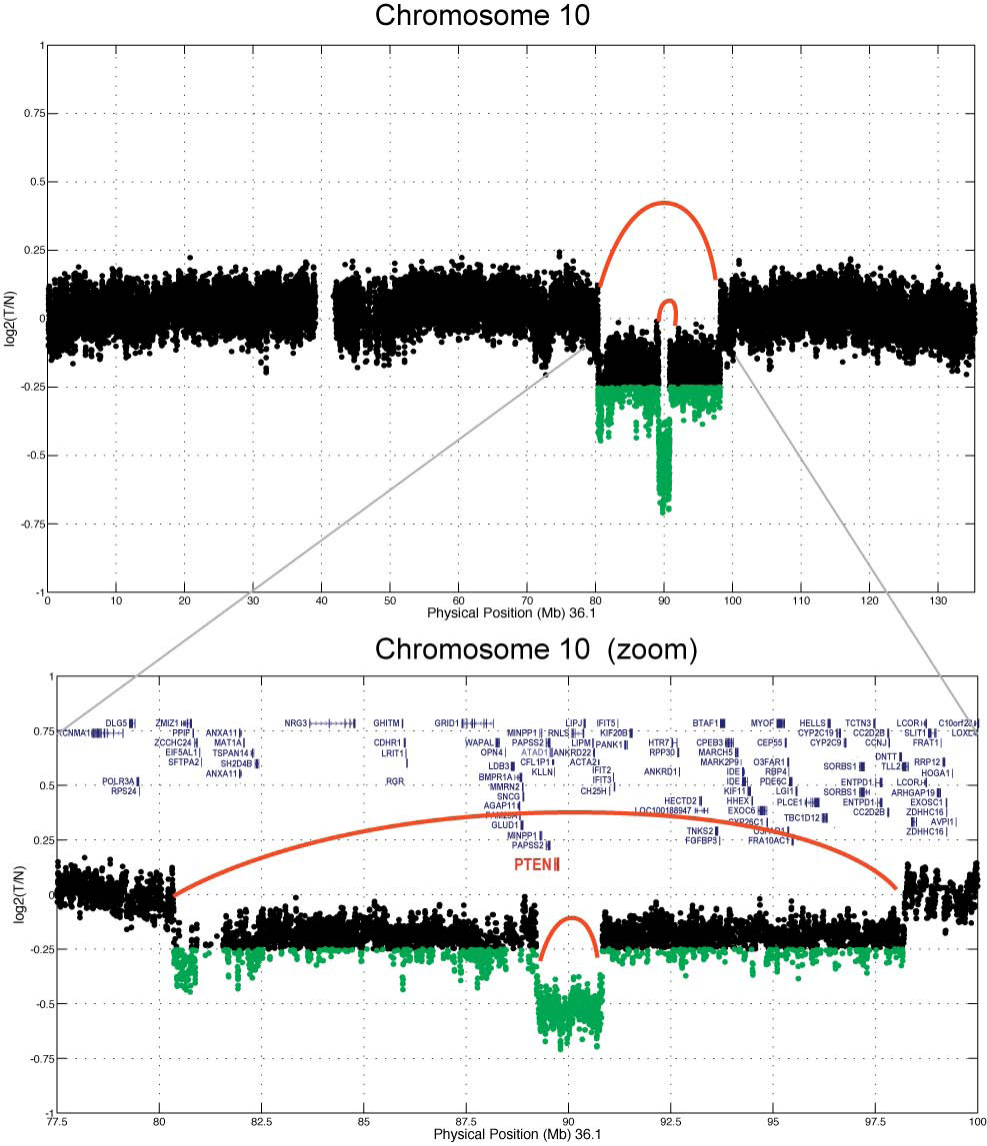
**Zoom in of the 10q region containing focal homozygous deletion encompassing the *PTEN *tumor suppressor gene**.

To identify regions of somatic copy number loss, we utilized a basic algorithm that determined log2 ratios in coverage difference between tumor and germline over a sliding window of 4,000 bp. Regions of copy number gain or loss are shown in Figure 1. This tumor exhibited whole chromosome copy number gains of chromosomes 2 and 8, along with copy number loss of chromosome 19. Of most significance was an approximate 20 Mb interstitial deletion at 10q23, which also contained a more focal region (2 Mb) of homozygous loss that encompassed the *PTEN *tumor suppressor gene (Figure 2). No other regions of focal gain or amplification were detected in this tumor (validation data are presented in Additional file 4).

To identify potential *cis *chromosomal rearrangements and translocation events, we searched for significant evidence of discordant mate pairs. The long insert mate pairs provide improved power for detecting structural alterations through improved clonal coverage. Clonal coverage can be defined as the genomic coverage (that is, 30×) multiplied by the length of the insert (1,500 bp), divided by the amount of sequence derived from each mate pair (100 bp). For example, at 37× genomic coverage for our tumor specimen and with 1,500 bp average mate-pair insert size, and with 2 × 50 bp mate-pairs (or 100 bp total), we achieve a clonal coverage of 432×. With such high clonal coverage we have significant power to detect evidence of discordant mate-pair reads, where the length of the insert deviates substantially from the mean insert length and/or map to different chromosomes or chromosomal regions. Utilizing an algorithm that identified discordant mate-pairs specific to the tumor, we discovered two independent translocation events occurring in the tumor. Both events involve genes on each side of the translocation event. One event is evidenced by significant discordant read pairs in the tumor mapping to the *LINGO2 *(leucine rich repeat and Ig domain containing 2) locus at 9q21.1 (chr9: 27990017-27991975), which is translocated to the *TTC28 *(tetratricopeptide repeat domain 28) locus at 22q12.1 (chr22: 27401302-27401562) (Figure 1). A second event is evidenced by discordant mate-pair read mapping to the *PRIM2 *(primase, DNA, polypeptide 2) locus at 6p12.1 (chr6: 57450028- 57451992) and to the *NPAS3 *(neuronal PAS domain protein 3) locus at 14q13.1 (chr14: 33206124- 33207653) (Figure 1).

## Discussion

Adenocarcinomas of the ampulla of Vater are relatively rare, accounting for only 0.2% of gastrointestinal cancers [17]. Perhaps due to their location and propensity to present with jaundice at an early resectable stage, these tumors are more likely to be resectable at the time of diagnosis than are pancreatic cancers [18]. Furthermore, in comparison to pancreatic cancer, resected ampullary cancers are associated with better 5-year survival rates of 34 to 61% [19-21]. Surgical series have demonstrated the factors affecting survival include completeness of surgical resection and nodal status. Surgical treatment for ampullary cancer and cancers in the head of the pancreas are similar in that surgeons perform a pancreaticoduodenectomy. Thereafter, the treatments may diverge. There is no clear consensus on the role of or the optimal regimen for adjuvant chemotherapy in ampullary cancers. Similarly, in part due to its relative rarity, there is no clear standard chemotherapeutic regimen for recurrent or metastatic ampullary cancer.

A better understanding of molecular oncogenesis and the emergence of targeted agents will likely lead to improved treatment outcomes in this and other cancers. Our study used whole genome sequencing to analyze the genome of a resected ampullary carcinoma. We found expected as well as novel aberrations. We found an activating mutation in *KRAS *codon 12. *KRAS *mutations are common in ampullary cancer although the 25 to 37% incidence appears to be lower than the approximately 95% rate of *KRAS *mutation seen in pancreatic adenocarcinomas [13,14,22,23]. Furthermore, similar to what is seen in colonic adenomas, *KRAS *mutations occur in benign ampullary adenomas, suggesting activating mutations of *KRAS *are relatively early events in the progression toward cancer and the mutation does not appear to affect prognosis [14]. This tumor also demonstrated a somatic nonsynonymous mutation in *SMAD4 *(mothers against decapentaplegic homolog 4), which has been observed previously in 50% of ampullary cancers but infrequently in bile duct cancers [24].

The most notable gene deletion we found was a focal deletion of a region in chromosome 10 including the *PTEN *tumor suppressor gene (phosphate and tensin homologue deleted on chromosome 10). Cowden's syndrome is characterized by a germline mutation in the *PTEN *gene resulting in loss of function. This syndrome is characterized by noncancerous hamartomas of the skin and mucous membranes and affected patients have in increased risk of tumors of the breast, thyroid, uterus and gastrointestinal tract. Benign tumors of the ampulla of Vater have been reported in patients with Cowden's syndrome but are not a common feature within cancers of the ampulla. Loss of PTEN expression by immunohistochemisty has been associated with liver metastases and poor prognosis in colon cancer [25]. In a large-scale survey of the genomic aberrations of pancreatic cancers, *PTEN *deletions were not seen, although small deleterious coding mutations were detected [26]. We can conclude that despite their anatomic location in proximity to the pancreas, ampullary cancers are distinct entities from adenocarcinoma of the pancreas and bile duct cancers and thus should be treated as a different entity.

**Figure 3:**
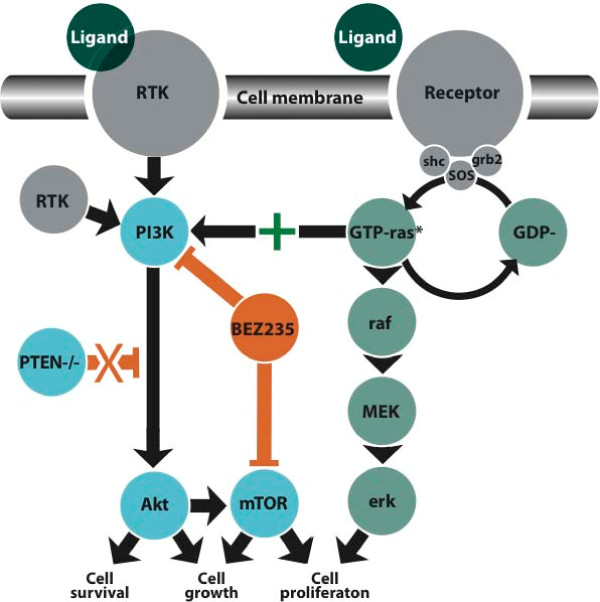
Simplified map and interactions of the phosphoinositide 3-kinase (PI3K) and RAS pathways highlighting the genomic aberrations (-, loss of function mutation, *gain of function mutation) identified in a cancer of the ampulla of Vater and the putative therapeutic site of vulnerability. ERK, extracellular-signal-regulated kinase; grb2, growth factor receptor bound protein 2; mTOR, mammalian target of rapamycin; RTK, receptor tyrosine kinase; SHC, SHC (Src homology 2 domain containing) transforming protein 1; SOS, son of sevenless.

To that end, the loss of *PTEN *expression is important not only in the pathogenesis but because it exposes a potential therapeutic target (Figure 3). The *PTEN *protein product is an inhibitor of phosphoinositide 3-kinase (PI3K) and downstream signaling through AKT. Phosphorylation of Akt results in phosphorylation of several target proteins involved in regulation of key cellular functions, including cell proliferation, glucose metabolism, protein translation, and cell survival [27]. Additionally, activation of the PI3K pathway has been linked to activation of mammalian target of rapamycin (mTOR), although the mechanism is not yet fully elucidated [28]. The presence of a deletion in *PTEN *in this ampullary cancer would be predicted to release from inhibition activation of the PI3K/mTOR pathway. Consequently, one can infer that an agent that is a dual PI3K/mTOR inhibitor, such as NVP-BEZ235, would be an attractive therapeutic option for our patient should his disease recur [29]. NVP-BEZ235 and other agents like it have been shown *in vitro *to inhibit growth of cancer cells with activating mutations of PI3K and are all under clinical development [30]. In the case presented here, however, the tumor carries both a *KRAS *activating mutation and complete inactivation of *PTEN*, supporting dual activation of both the MEK/ERK and the PI3K/AKT axes (Figure 3). The inhibition of only one axis may not be sufficient for effective treatment as there is likely to be compensatory activity from the other activated axis.

Our group reported the beneficial results seen in a clinical trial on patients with refractory solid tumors whose chemotherapy was chosen based on analysis of tumor biopsies using immunohistochemistry and expression arrays [31]. New technologies such as applied herein have made high-throughput whole-genome sequencing a more rapid and cost-effective process in a manner not possible with older technologies such as Sanger sequencing. The prospect is raised, therefore, that one may soon be able to apply whole-genome sequencing to the analysis of an individual patient's tumor to guide an informed choice of a therapeutic regimen. This type of personalized or precision medicine has only begun to be studied. Several limitations remain before this whole-genome sequencing methodology can be widely applied, including the need for improved and standardized bioinformatic analysis, along with reliable and rapid methods for validation of genomic findings and cost. Furthermore, if a target is found, one must have access to an agent and, in many cases, such agents may not be approved for clinical use. Thus, we must begin to understand the links between genomic profile and drug context in early drug development. This is amplified even more where there is evidence to support combination therapies.

## Conclusions

We have analyzed the whole genome sequence of a cancer of the ampulla of Vater to uncover the compendium of somatic events occurring in this tumor. Among the mutations discovered were those that might be considered potential therapeutic vulnerabilities. As whole-genome sequencing becomes more rapid and less expensive, the potential for targeted and truly personalized treatments increases. Consequently, as we continue to refine our abilities to uncover the full landscape of somatic alterations, we must in parallel continue innovative drug development methods, including preclinical and early phase I combination trials. This will allow us to understand toxicities and appropriate dosing regimens, to obtain the safest and most appropriate combinations matched to specific genomic and molecular contexts.

## Abbreviations

BAM: binary sequence alignment map; bp: base pair; mTOR: mammalian target of rapamycin; PCR: polymerase chain reaction; PI3K: phosphoinositide 3-kinase; SNP: single nucleotide polymorphism; WIRB: Western Institutional Review Board.

## Competing interests

The authors declare that they have no competing interests.

## Authors' contributions

MJD, DWC, DVH, and JC conceived of the study, and participated in its design and coordination, and drafted the manuscript. LK and JT participated in study design and coordination. SS participated in the sequence alignment and manuscript drafting. TMM, JD, and IC carried out molecular genetic and sequencing studies. AC, TI, and JA participated in sequence alignment. AB, AW, AD, DL, GH and LK participated in sample acquisition, sample quality assessment, and preparation. All authors read and approved the final manuscript.

## Supplementary Material

Additional file 1 **Supplemental methods giving additional details regarding the methods used for the detection of somatic translocations and intrachromosomal rearrangements**. Click here for file

Additional file 2 **Validation primer sets: sequencing and quantitative PCR primer pairs used in validation of next generation sequencing results**. Click here for file

Additional file 3 **Figure showing the results of validation by Sanger sequencing: validation of single nucleotide variants (n = 10) and a local deletion**. Sequencing electropherograms depicting specific mutations in selected genes in forward and reverse orientations. Click here for file

Additional file 4 **Figure demonstrating the validation of the next generation sequencing comparative genomic hybridization findings of a *PTEN *deletion**. Results depicted show the relative fold changes measured by quantitative PCR in tumor versus normal genomic DNA in specific regions of chromosome 10q affected by hemi- (*RGR *and *HHEX*) or homozygous (*PTEN*) deletion. Click here for file
