# Supplementary material for: Cancer of the ampulla of Vater: analysis of the whole genome sequence exposes a potential therapeutic vulnerability
Source: Genome Med. 2012 Jul 4;4(7):56. doi: 10.1186/gm357 (PMC3580412; doi:10.1186/gm357)
Supplement: Additional file 1 — Supplemental methods giving additional details regarding the methods used for the detection of somatic translocations and intrachromosomal rearrangements. [file gm357-S1.DOCX]

**Somatic detection of translocations and intrachromosomal rearrangements.**

Identification of candidate intra- and inter-chromosomal alterations occurred as follows within a perl script in the section below.

First a soft sliding window analysis of 2Kb is conducted. A hard window is given by some padding on each side given by the user (500 bp for this analysis). The following information is collected from both the tumor and the normal sample: Reads with unmapped mates or low mapping quality (less than 10) that are filtered total number of reads aligned in each window. We determine two insert size cutoffs by employing an outlier detection method is employed on the tumor sample. Normality is assumed for the distribution of insert sizes for the chromosome. Under this assumption a cutoff of 3 times the 5% trimmed standard deviation on either side of the 5% trimmed mean is determined. At the lower end cutoff, a minimum coverage can also be specified by the user (see code below). The maximum of this minimum coverage or the minimum cutoff serves as the new lower cutoff. All read pairs with insert size outside this interval are considered “discordant”.

For all windows in the normal sample, we now determine the proportion of discordant read pairs. Again under assumption of normality an upper cutoff specified by trimmed mean plus standard deviation is determined and this value serves as the tolerance parameter for the underlying statistical model. The user who can specify a given value of tolerance by fiat can override this value.

To remove regions of possible artifacts, we employ the same strategy as in insert sizes to the distribution of aligned reads in the window from the tumor data. An outlier window with too little or too many aligned reads in the tumor is eliminated from further consideration.

For each remaining window we now determine the number of discordant pairs mapped in tumor and normal sample and the total aligned reads. A statistical model then determines the probability that the enrichment of discordant reads in the tumor is not somatic. Approximating the binomial distribution of proportions by the Normal distribution for each sample and then taking their z-scores do this. A p-value then is the probability of the negative of the absolute value of the difference of these two z-scores plus a factor that determines that the event is somatic. The p-value is the probability of the region being not somatically enriched.

Now the factor that determines whether the event is somatic is essentially the difference of two percentages, the proportion of discordant reads bigger than 10% (or any user specified cutoff) in the window from tumor sample and the proportion of discordant reads bigger than tolerance in the normal window multiplied by a log penalty function.

**B. Script for detection of candidate translocations.**

#!/usr/bin/perl -w

#

# Program: translocations.pl

# Author: Shripad Sinari

# Created: 2011-06-13

#

use strict;

use warnings;

use Getopt::Long;

use IO::File;

use Data::Dumper;

use Time::HiRes;

use Pod::Usage;

use Math::CDF qw(:all);

use Time::localtime;

use List::Util qw(first max maxstr min minstr reduce shuffle sum);

use Statistics::Descriptive;

use Statistics::Robust::Scale qw(MAD);

use vars qw( $CVSID $REVISION $VERBOSE $VERSION %PARAMS );

( $REVISION ) = '$Revision: 1.11 $ ' =~ /\$Revision:\s+([^\s]+)/;

( $CVSID ) = '$Id: processPSL.pl,v 1.11 2009/02/25 23:25:32 ssinari Exp $'

=~ /\$Id:\s+(.*)\s+/;

### Assumptions:

## 1: Bam is split by Chromosome

## 2: Bam is sorted

MAIN: {

# Setup defaults for important variables.

$|++;

my $SAMTOOLSBIN="/Users/ssinari/tools/samtools-0.1.7a/samtools";

#my $SAMTOOLSBIN="/packages/samtools-0.1.12/samtools";

#my $SAMTOOLSBIN="/home/ssinari/tools/samtools-0.1.7a/samtools";

my $tumorFile = '';

my $normalFile = '';

my $outputFile = '';

my $start = 1;

my $windowsize = 2000;

my $mappingQuality = 10;

my $coverage = 10;

my $chr = [];

my $dosage = 1;

my $totalreads = 0;

my $totalcoverage = 0;

my $ctotalreads = 0;

my $ctotalcoverage = 0;

my $cutoff = 0.05;

my $readlength = 50;

$VERBOSE = 0;

$VERSION = 0;

my $help = 0;

my $man = 0;

pod2usage(1) unless (scalar @ARGV > 0);

my $results = GetOptions (

't|tumor=s' => \$tumorFile, # -t

'n|normal=s' => \$normalFile, # -n

'o|output=s' => \$outputFile, # -o 's|samtools=s' => \$SAMTOOLSBIN, # -s

'c|chromosomes=s@' => $chr, # -c

'f|cutoff=f' => \$cutoff, # -f

'w|window=i' => \$windowsize, # -w 'q|mappingQuality=i' => \$mappingQuality, # -q

'd|dosage=f' => \$dosage, # -d

'a|coverage=i' => \$coverage, # -a

'v|verbose+' => \$VERBOSE, # -v

'version!' => \$VERSION, #

'h|help|?' => \$help, # -h

'm|man' => \$man # -m

);

pod2usage(1) if $help;

pod2usage(-exitstatus => 0, -verbose => 2) if $man;

if ($VERSION) { print "$CVSID\n"; exit }

pod2usage(1) if $tumorFile eq '';

pod2usage(1) if $normalFile eq '';

pod2usage(1) if $outputFile eq '';

if (! -r $SAMTOOLSBIN){print STDERR "$SAMTOOLSBIN is not readable\n";exit;}

my $logFile = $outputFile;

$logFile=~s/gff$/log/;

open (OUTFILE,"> $outputFile") || die "cannot open $outputFile for writing:$!\n";

open (LOGFILE,"> $logFile") || die "cannot open $logFile for writing:$!\n";

my $cmdline = ctime()." ".$0 .' '. join(' ', @ARGV)."\n";

print LOGFILE $cmdline;

my $time = time();

my $minAligned = int($coverage*$windowsize/$readlength);

open (THFILE,"$SAMTOOLSBIN view -H $tumorFile |") || die "cannot open $tumorFile for reading:$!\n";

my %tsq = ();

while (<THFILE>){

next unless $_=~/^\@SQ/;

$_=~/SN:(\w+)\s+LN:(\d+)/;

$tsq{$1}=$2;

}

close THFILE;

open (NHFILE,"$SAMTOOLSBIN view -H $normalFile |") || die "cannot open $normalFile for reading:$!\n";

my %nsq = ();

while (<NHFILE>){

next unless $_=~/^\@SQ/;

$_=~/SN:(\w+)\s+LN:(\d+)/;

$nsq{$1}=$2;

}

close NHFILE;

foreach my $sq ( sort {$a cmp $b} keys %tsq){

if (! exists $nsq{$sq}){print STDERR "Sequence $sq is not part of Normal bam\n"; exit;}

}

foreach my $sq ( sort {$a cmp $b} keys %nsq){

if (! exists $tsq{$sq}){ print STDERR "Sequence $sq is not part of Tumor bam\n"; exit;}

}

print LOGFILE "The sequences aligned between Tumor and Normal bams \n";

my @sq = ();

@sq = @{$chr} if defined $chr && $chr ;

@sq = split(/,/,join(',',@sq));

foreach my $c ( sort {$a cmp $b} @sq){

if (! exists $nsq{$c}){ exit;}

}

@sq = sort { $a cmp $b } keys %nsq if ! @sq;

#print join(":",@sq),"\n";exit;

my $REF = join(",",@sq);

print LOGFILE

"Initial parameters:

Tumor: $tumorFile

Normal: $normalFile

Output: $outputFile

Dosage : $dosage

Reference: $REF

Window: $windowsize

Min Aligned: $minAligned

MaqQ: $mappingQuality

###################################\n";

print

"Initial parameters:

Tumor: $tumorFile

Normal: $normalFile Output: $outputFile

Dosage : $dosage

Reference: $REF

Window: $windowsize

Min Aligned: $minAligned

MaqQ: $mappingQuality

###################################\n";

print LOGFILE "Initiating the search for discordant regions\n";

my $trCount=1;

foreach my $ref(@sq){

my $refLen = $nsq{$ref};

print LOGFILE "Now processing $ref ...\n";

open (TFILE,"$SAMTOOLSBIN view $tumorFile $ref |")
 my $linecount = 0;

my $end = $windowsize;

#### Collect data by bins of length windowsize ###

my %br1 = ();

my %br2 = ();

my %discordant = ();

my %aligned = ();

print LOGFILE "TWindow: ",$end,"\n";

my $i=0;

while (<TFILE>){

$linecount++;

next if $_=~/^@/;

chomp;

my $qname,$flag,$rname,$pos,$mapq,$cigar,$mrnm,$mpos,\
$isize,$strand,$seq,$qual,$opt) = split(/\t/,$_);

next if $mapq < $mappingQuality;

next unless rand() <= $dosage;

$totalreads+=1;

if ($pos <= $end){

$aligned{$end}++;

}else{

$end += $windowsize;

$end = $refLen if $end > $refLen;

$aligned{$end}++;

}

next if ($rname ne $ref) or ($mrnm eq "*") or ($mrnm eq "=");

push @{$br1{$end}{$mrnm}},$pos; ### Collects pos of read 1 aligning to the ref

push @{$br2{$end}{$mrnm}},$mpos;

$discordant{$end}{$mrnm}++;

}

close TFILE;

print LOGFILE "Window: ",$end,"\n";

####---####

my %cbr1 = ();

my %cbr2 = ();

my %cdiscordant = ();

my %caligned = ();

$end = $windowsize;

open (NFILE,"$SAMTOOLSBIN view $normalFile $ref |");
print LOGFILE "NWindow: ",$end,"\n";

#### Collect data by bins of length windowsize ###

$i=0;

while (<NFILE>){

$linecount++;

if( 0 == ($linecount % 1000000) ){

}

next if $_=~/^@/;

chomp;

my ($qname,$flag,$rname,$pos,$mapq,$cigar,$mrnm,$mpos,$isize,$strand,\
 $seq,$qual, $opt) = split(/\t/,$_);

next if $mapq < $mappingQuality;

next unless rand() <= $dosage;

$ctotalreads+=1;

if ($pos <= $end){

$caligned{$end}++;

}else{

$end += $windowsize;

$end = $refLen if $end > $refLen;

$caligned{$end}++;

#print "$end\n";

}

next if ($rname ne $ref) or ($mrnm eq "*") or ($mrnm eq "=");

push @{$cbr1{$end}{$mrnm}},$pos; ### Collects pos of read 1 aligning to the ref

push @{$cbr2{$end}{$mrnm}},$mpos;

$cdiscordant{$end}{$mrnm}++;

}

close NFILE;

$totalcoverage = ($totalreads*$readlength/$refLen);

$ctotalcoverage = ($ctotalreads*$readlength/$refLen);

print LOGFILE "Run Dosage: $dosage TReads : $totalreads TCoverage : $totalcoverage CReads : $ctotalreads CCoverage : $ctotalcoverage\n";

### Find the top hit for each bin #####

my @intervals = sort { $a <=> $b } keys %discordant;

%{$discordant{$intervals[$_]}})," A:",$aligned{$intervals[$_]},"\n" foreach (0..$#intervals);

%{$discordant{$intervals[$_]}} )," A:",$aligned{$intervals[$_]},"\n" foreach (0..$#intervals);

print LOGFILE "Finding top hit\n";

my @hit = ();

foreach $end (@intervals) {

my @sort = sort { $discordant{$end}{$b} <=> $discordant{$end}{$a} } keys %{$discordant{$end}};

push @hit , shift @sort;

}

### Private methods for calculating trimmed stddev ######

my $stat_x = Statistics::Descriptive::Full->new();

my $stat_xx = Statistics::Descriptive::Full->new();

sub add_x {

$stat_x->add_data( @_ );

$stat_xx->add_data( map {$_*$_} @_ );

}

sub trimmed_stddev {

my $lower = shift;

my $upper = @_ ? shift : $lower;

my $trm_x = $stat_x->trimmed_mean( $lower, $upper );

my $trm_xx = $stat_xx->trimmed_mean( $lower, $upper );

my $nn = int( $stat_x->count() * ( 1.0 - $lower - $upper ) );

return ($nn <= 1 ? 0.0 : sqrt(($trm_xx - $trm_x * $trm_x ) * $nn / ($nn -1)) );

}

#####---------------------------------------####

my @cprop = ();

foreach (0..$#intervals) {

my $cd = $cdiscordant{$intervals[$_]}{$hit[$_]} || 0;

my $ca = $caligned{$intervals[$_]} || 1;

push @cprop, $cd/$ca;

}

my $s=Statistics::Descriptive::Full->new();

$s->add_data(@cprop);

#my $sm = $s->trimmed_mean(0.05) || 0;

#add_x(@cprop);

#my $smad = trimmed_stddev(0.05) || 0;

my $sm = $s->median() || 0;

my $smad = MAD(\@cprop) || 0;

my $tol = $sm + $smad;

my $tolerance = min(($tol,0.05));

print LOGFILE ctime()," MED:$sm MAD:$smad TOL:$tolerance\n";

my @prop = ();

foreach (0..$#intervals) {

my $td = $discordant{$intervals[$_]}{$hit[$_]} || 0;

my $ta = $aligned{$intervals[$_]} || 1;

push @prop, $td/$ta;

}

if ($cutoff=~/[cC]/){

$s->add_data(@prop);

my $pmean = $s->trimmed_mean(0.05) || 0;

add_x(@prop);

my $psd = trimmed_stddev(0.05) || 0;

$cutoff = $pmean + 3*$psd;

print LOGFILE "M:",$pmean," SD:",$psd," ";

}

print LOGFILE "CUT:$cutoff\n";

my $Rstat = Statistics::Descriptive::Full->new();

my %probs = ();

my %score = ();

foreach (0..$#intervals) {

if ($aligned{$intervals[$_]} > $minAligned) {

#my $cd = $cdiscordant{$ref}{$intervals[$_]}{$hit[$_]} || 0;

#my $ca = $caligned{$ref}{$intervals[$_]} || 1;

my $cd = $cdiscordant{$intervals[$_]}{$hit[$_]} || 0;

my $ca = $caligned{$intervals[$_]} || 1;

my $td = $discordant{$intervals[$_]}{$hit[$_]} || 0;

my $ta = $aligned{$intervals[$_]} || 1;

my $prob = 1;

$prob = probability($td,$ta,$cd,$ca,$tolerance,\
 $cutoff) if ($ca > $minAligned);

$probs{$intervals[$_]} = $prob;

$score{$intervals[$_]} = 1000;

$score{$intervals[$_]} = -10*log($prob)/log(10) if $prob; #

my ($rl,$ru) = @{findEnds($br1{$intervals[$_]}{$hit[$_]})};

my ($mb,$me) = @{findEnds($br2{$intervals[$_]}{$hit[$_]})};

my $rR = $ru - $rl;

my $mR = $me - $mb;

my $cprop = $cd/$caligned{$intervals[$_]};

my $tprop = $td/$aligned{$intervals[$_]};

my $tscore = $score{$intervals[$_]};

my $p = $probs{$intervals[$_]};

print LOGFILE "I: $intervals[$_] tD: $td tA: $ta P: $tprop \

cD: $cd cA: $ca P: $cprop PROB: $p SCORE: $tscore\

rR: $rR RS: $rl RE: $ru H : $hit[$_] MS: $mb ME: $me\n";

if ( defined $probs{$intervals[$_]} && $probs{$intervals[$_]}
 < 0.05 && $rR > 500 && $mR < 20000){

my $fref = $ref;

my $fmrnm = $hit[$_];

my $trID = "tr".$trCount;

print LOGFILE "SOMATIC I: $intervals[$_] tD: $td tA: $ta P: $tprop cD: $cd cA: $ca P: $cprop PROB: $p SCORE: $tscore rR: $rR RS: $rl RE: $ru H : $hit[$_] MS: $mb ME: $me\n";

print OUTFILE join("\t",($fref,$trID, "TR", $rl, $ru,$score{$intervals[$_]}, ".", ".", "region=$fmrnm:$mb-$me")

),"\n";

$trCount++;

}

}

}

}

print LOGFILE ctime(),"\n";

print LOGFILE "Done.\n";

close LOGFILE;

close OUTFILE;

}

sub findEnds{

my $r = shift;

my $mstat = Statistics::Descriptive::Full->new();

$mstat->add_data($r);

my $mm = $mstat->median();

my $misd = MAD($r);

my $ml = int($mm - $misd);

my $mu = int($mm + $misd);

return [$ml,$mu];

}

sub probability{

my ($y1,$n1,$y2,$n2,$tolerance,$cutoff) = @_;

my $prob = 1;

if ($n1 && $n2){

my $p1 = $y1/$n1;

my $p2 = $y2/$n2;

my $penalty = log(1+$y2);

my $tmean = $n1*$p1;

my $tstd = sqrt($n1*$p1*(1-$p1)) || 1;

my $nmean = $n2*$p2;

my $nstd = sqrt($n2*$p2*(1-$p2)) || 1;

my $ztumor = ($p1-$tmean)/($tstd);

my $znormal = ($p2-$nmean)/($nstd);

my $intolerance = $penalty*($p2-$tolerance)*100;

my $sig_cutoff = ($p1-$cutoff)*100;

$prob = pnorm(($ztumor-$znormal+$intolerance-$sig_cutoff));

$prob=$prob**0.1;

}

return $prob

}

__END__

=head1 NAME

translocations.final.pl - discovers interchromosomal translocations

=head1 SYNOPSIS

translocations.final.pl [options] -t TUMOR.bam -n NORMAL.bam -o OUTPUT.gff -a MINIMUM_COVERAGE -c CHROMOSOMES

=head1 OPTIONS

-t | --tumor tumor bam file

-n | --normal normal bam file

-o | --output output file name

-s | --samtools location of the samtools binary

-w | --window window size to be used for calculations

-a | --coverage minimum coverage required in the window. Defaults to 10,

meaning atleast 10X coverage

-d | --dosage Used for downsampling. The sample will be downsampled to

the value provided to this option

-f | --cutoff set cutoff threshold for expected proportion of discordant reads

-q | --mappingQuality minimum mapping quality of the reads to be considered (default to 10)

-v | --verbose print progress and diagnostic messages

--version print version number

-h | --help display help

-m | --man display man page

=head1 AUTHOR

=over

=item Shripad Sinari, L<mailto:ssinari@tgen.org>

=back

=head1 COPYRIGHT

This software is copyright 2011 by The Translational Genomics Research

Institute. All rights reserved. This License is limited to, and you

may use the Software solely for, your own internal and non-commercial

use for academic and research purposes. Without limiting the foregoing,

you may not use the Software as part of, or in any way in connection

with the production, marketing, sale or support of any commercial

product or service or for any governmental purposes. For commercial or

governmental use, please contact licensing@tgen.org. By installing this

Software you are agreeing to the terms of the LICENSE file distributed

with this software.

In any work or product derived from the use of this Software, proper

attribution of the authors as the source of the software or data must be

made. The following URL should be cited:

L<http://bioinformatics.tgen.org/software/solexa/>

=cut
