# Supplementary material for: Cancer of the ampulla of Vater: analysis of the whole genome sequence exposes a potential therapeutic vulnerability
Source: Genome Med. 2012 Jul 4;4(7):56. doi: 10.1186/gm357 (PMC3580412; doi:10.1186/gm357)
Supplement: Additional file 3 — Figure showing the results of validation by Sanger sequencing: validation of single nucleotide variants (n = 10) and a local deletion. Sequencing electropherograms depicting specific mutations in selected genes in forward and reverse orientations. [file gm357-S3.PDF]

Supplementary Figure 1. Validation of single nucleotide variants (n=10) and a local deletion. Sequencing electropherograms depicting specific mutations in selected genes in forward and reverse orientations.

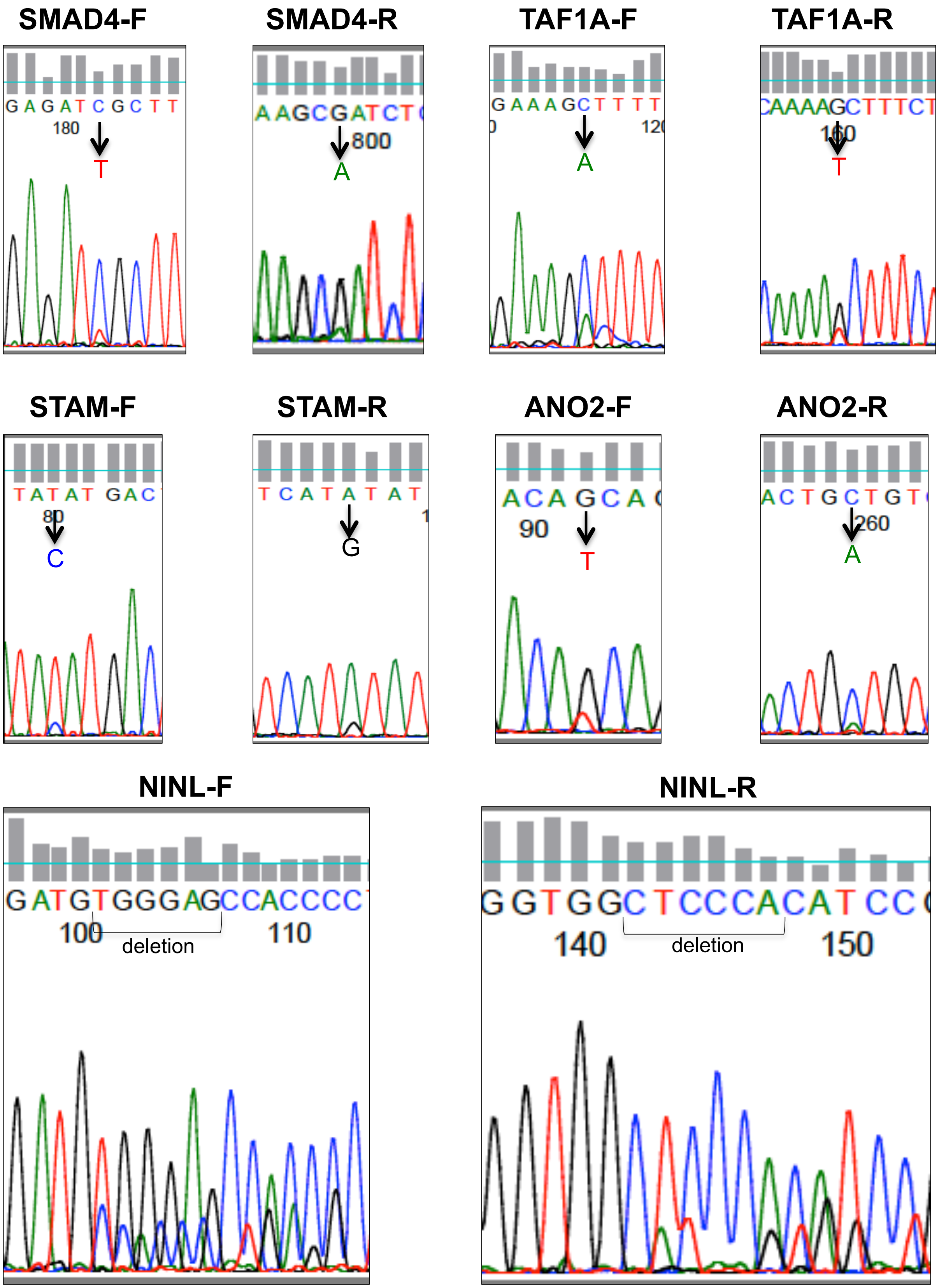

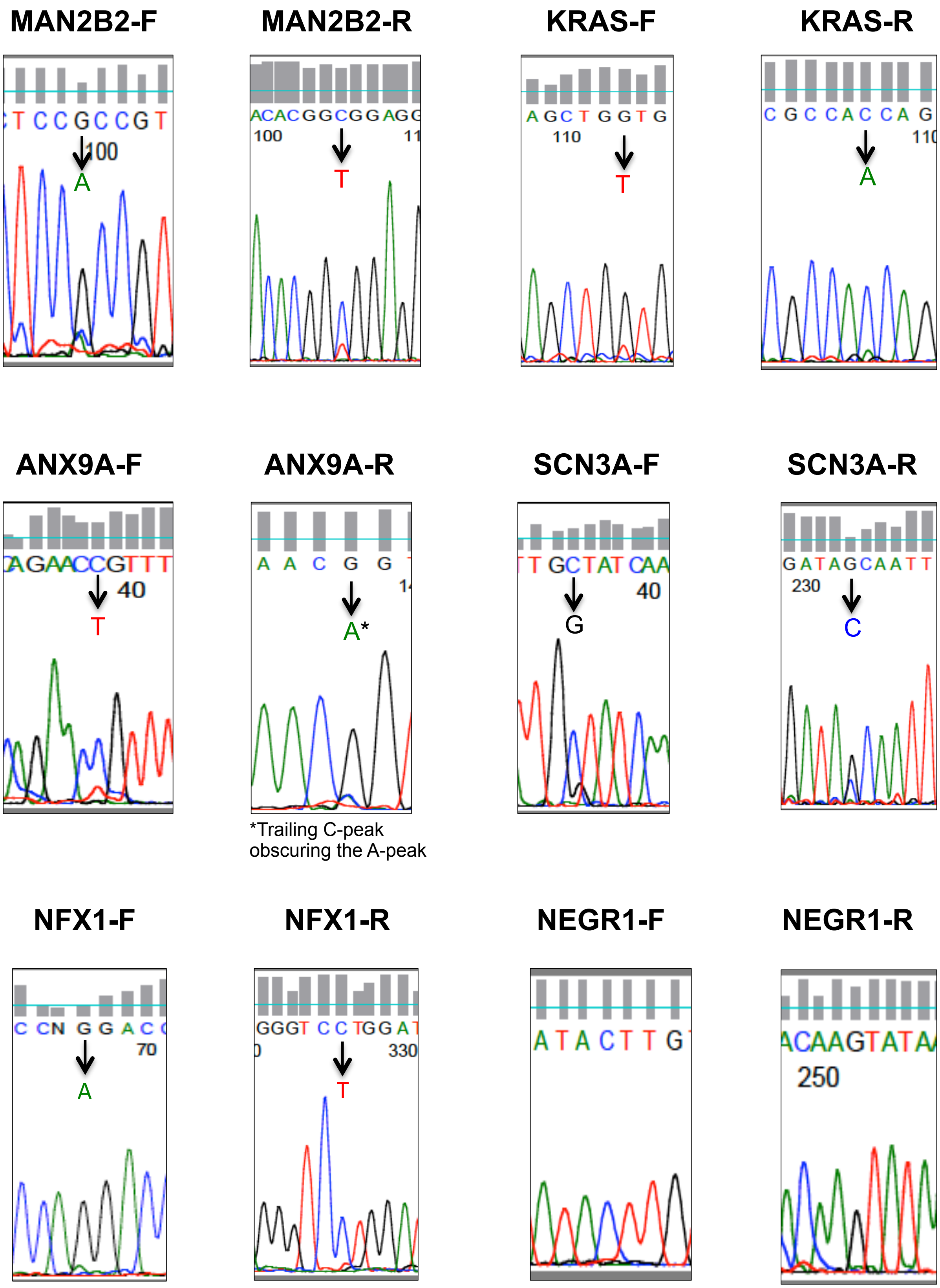

MAN2B2-F
A
MAN2B2-R
T
KRAS-F
T
KRAS-R
A
ANX9A-F
T
ANX9A-R
A*
SCN3A-F
G
SCN3A-R
C
NFX1-F
A
NFX1-R
T
NEGR1-F
NEGR1-R
*Trailing C-peak obscuring the A-peak
