# Supplementary material for: Cancer of the ampulla of Vater: analysis of the whole genome sequence exposes a potential therapeutic vulnerability
Source: Genome Med. 2012 Jul 4;4(7):56. doi: 10.1186/gm357 (PMC3580412; doi:10.1186/gm357)

Supplementary Figure 2. Validation of NextGen CGH findings for PTEN deletion. Relative fold changes measured by qPCR in tumor vs. normal genomic DNA in specific regions of chromosome 10q affected by hemi- (*RGR* and *HHEX*) or homozygous (*PTEN*) deletion. Rough coordinates of each gene are indicated on the schematic view of the chromosome; M=million base pairs.

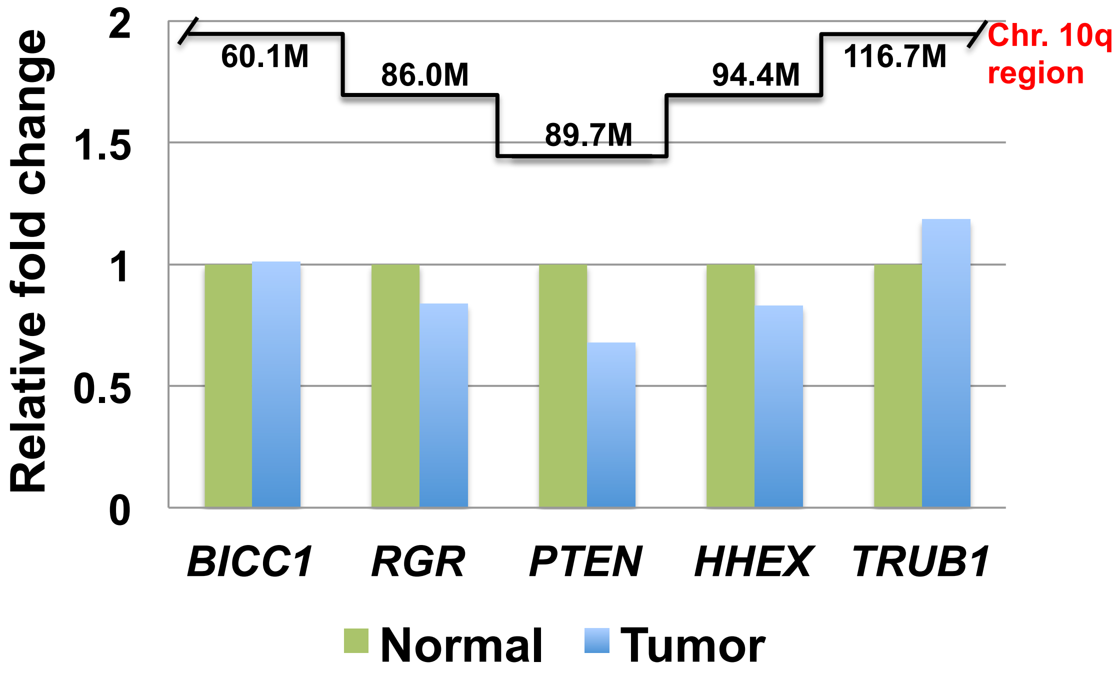

Supplement: Additional file 4 — Figure demonstrating the validation of the next generation sequencing comparative genomic hybridization findings of a PTEN deletion. Results depicted show the relative fold changes measured by quantitative PCR in tumor versus normal genomic DNA in specific regions of chromosome 10q affected by hemi- (RGR and HHEX) or homozygous (PTEN) deletion. [file gm357-S4.PDF]
